# Clinical Outcomes of Unrelated Umbilical Cord Blood Graft vs. Haploidentical Donor Transplantation: Critical Issues for an Adequate Comparison

**DOI:** 10.3389/fmed.2021.749810

**Published:** 2021-10-28

**Authors:** Diana Vanegas, Laura Niño-Quiroga, Mauricio Chaparro, Bernardo Camacho-Rodríguez, Marcela Estupiñán, Ana-María Perdomo-Arciniegas

**Affiliations:** ^1^Cord Blood Bank, Instituto Distrital de Ciencia, Biotecnología e Innovación en Salud, Bogota, Colombia; ^2^Unidad de Trasplante, Fundación HOMI-Hospital de la Misericordia, Bogota, Colombia

**Keywords:** hematopoietic stem cell transplantation, cord blood, haploidentical, HLA matching, clinical outcomes

## Abstract

Unrelated umbilical cord blood (UCB) and haploidentical grafts have been used for allogeneic hematopoietic stem and progenitor cell (HSPC) transplantation in patients without a related or non-related human leukocyte antigen (HLA)-matched donor. The less stringent HLA-matching requirement in both sources raises an important possibility for patients in need of urgent transplantation to treat any hematological disease. Selection of the best alternative donor is a difficult task that will depend on donor criteria, center experience, patient disease conditions, and risk, among others. Most comparisons available in scientific publications between both graft sources are obtained from retrospective analysis in wide time windows and a heterogeneous number of patients, types of disease, disease stages, previous treatments, graft source, conditioning regimen, graft vs. host disease (GVHD) approach, and evaluable endpoints. There is also an evident impact of the economic traits since low-income countries must consider less expensive treatments to satisfy the needs of the patients in the most effective possible path. Therefore, haploidentical transplantation could be an appealing option, even though it has not been completely established if any chronic treatment derived from the procedure could become a higher cost. In Colombia, there is a huge experience in UCB transplantation especially in units of pediatric transplantation where benign indications are more common than in adults. Due to the availability of a public UCB bank and HLA high-resolution typing in Colombia, there is a wider inventory of cord blood donors. Unfortunately, we do not have an unrelated bone marrow donor registry, so UCB is an important source along with haploidentical transplantation to consider in decision-making. This minireview focuses on comparing the main issues associated with the use of both HSCP sources and provides tools for physicians who face the difficult decision between these alternative donor sources.

## Introduction

Hematopoietic stem and progenitor cell (HSPC) transplantation is a potentially curative treatment that has been used for different disorders that affect hematopoiesis (1, 2). Depending on the disease, transplantation may be autologous when the HSPCs are obtained from the patient or allogeneic when the graft comes from a match-related or unrelated donor. Since the transplantation involves a bone marrow reconstitution in the patient, matching of human leukocyte antigen (HLA) between donor and recipient is probably the most considered and debating topic in the literature (3–6). However, according to some clinical practice guidelines, there are several additional factors that must be considered for the donor and HSPC source selection, such as the urgency of the transplantation, status and risk of the disease, donor criteria, and transplantation center experience (1, 7, 8).

It is known that only 30% of the patients who require an HSPC transplant have a fully HLA-matched sibling donor (6, 9, 10) and therefore, an alternative graft source, such as a haploidentical donor and UCB units, turns out very important for the clinical practice. However, there is huge variability in the clinical populations in which both graft sources are currently used, such as adult and pediatric patients, malignant and non-malignant disease, previous relapses or refractory disease, conditioning regimen, and prophylaxis treatment, among others. Therefore, we focused on verifying the information reported in the literature only for malignant disease in which umbilical cord blood and haploidentical transplantation are compared simultaneously as a two-arm design, retrospective or prospective, with overall survival or progression-free survival (PFS) evaluated as clinical endpoints and including information regarding the time of engraftment. Although there are several reports of the clinical results and experience with each graft source separately, we did not include them, considering the aforementioned variability.

There is an important experience in Colombia with UCB transplantation for pediatric patients and a higher inventory of cord blood donors with HLA high-resolution typing available in the public UCB bank which started 5 years ago (11). Since there is no unrelated bone marrow donor registry in the country, UCB turns out an important source along with haploidentical donors for patients who require transplantation. The purpose of this work is to verify the clinical outcomes in malignant disease treated with transplantation from these two sources and to consider the additional conditions that may impact not only the results but the selection of one source over the other.

## Haploidentical Donor Transplantation Rate Increase

Over the past 5 years, some authors have been pointing out an evident increase of haploidentical donor transplantation procedures and a significant decrease in UCB for allogeneic transplants in Europe and the United States (10, 12–14). In 2018, Weisdorf (14) reported that although the total number of HSPC transplantations per year in the US has been stable, UCB use has declined from 800 to 600 per year, while haploidentical has raised from 500 to 1,200 by the end of 2016. In the European scenario, UCB use has decreased from over 800 transplantations per year in 2010 to <500 in 2016, and it appears to be restricted to the pediatric population. Haploidentical transplantation numbers have been rising, mainly in the less wealthy countries, suggesting an economic effect on the donor selection process (12, 13).

There are some particularities in Latin America regarding this issue. Although HSPC transplantation has been performed for the last 50 years in Europe and the US (4), in Latin American countries, its practice barely started in the 1980s only in Brazil and Argentina and has been increasing from that moment in the other countries (15). However, the available information regarding the number of hematopoietic transplants performed in the 28 countries from the Latin American region is scarce and probably underestimated because of non-reporting transplantation centers (16). From the obtained data until 2012, Jaimovich et al. reported a 20% increase in allotransplants from 2009, mainly as a treatment for leukemia. There was also a relation between gross national income (GNI) per capita and transplantation activity, which was completely absent in countries with <3.3 million people or GNI per capita < US$3,400. However, the transplantation rate did not exhibit a clear trend related to GNI per capita only, and some high-income countries bear a low transplantation rate. It is, however, clear that by 2012, the Latin American transplant rate was from 5- to 8-fold lower than North America and Europe, and the density of transplant teams in proportion with the population was also lower, only considering data from the reporting countries, without any auditing (16).

There is also an underestimated genetic variability in most of the countries and regions in Latin America. The European colonization, African slavery, native American populations, and different migration processes from the Middle East and Asia have established a mixed gene pool in the region, including HLA genes, and therefore, probably impacting the search for a suitable allogeneic donor in European or North American Bone marrow (BM) donor registries. Registries available in Latin American countries are considerably smaller, the searching activities are less organized and take longer times, which is critical for patients with malignant diseases (16). Since haploidentical transplantation (T cell repleted, without any *ex vivo* manipulation) solves the problem of finding an HLA-matching donor and its associated high costs, it represents an appealing option in developing countries and its use is rapidly increasing, as reported in the US and Europe (17).

## Haploidentical Donor and Cord Blood Unit Selection Inequality

The main question would be whether haploidentical transplantation exhibits superiority in terms of clinical outcomes compared to UCB transplantation. As previously mentioned, a haploidentical donor could be guaranteed to almost every transplant candidate and it is economically beneficial. The first decades of haploidentical transplantation performance did not exhibit favorable clinical results, mainly due to the poor quality of life and high morbimortality rates in the patients, caused by the incidence of GVHD. These clinical problems were partially surpassed using *in vivo* and *ex vivo* T-cell depletion techniques (9), which altogether with higher cellular doses initially favored clinical results of HSPC transplantation of haploidentical donors compared to UCB grafts.

On the other hand, it had been stated that, unlike other sources, UCB did not require an allele-level matching in all five or six HLA loci (HLA-A, -B, -C, -DRB1, -DQB1, and DPB1) and that cell dose comprises a more impacting factor than HLA matching on clinical outcomes (3, 5). However, recent evidence suggests that allele-level HLA matching improves clinical outcomes using UCB (18–21). It is also a matter of concern that an important proportion of patients previously transplanted with UCB, matched by low-resolution typing in HLA-I, has one or more additional HLA mismatches when allele-level typing was retrospectively analyzed (22). Therefore, many UCB-transplanted patients with a previously stated 4/6 HLA match with the graft have in fact less than a 3/6. This is a clear disadvantage for UCB compared to haploidentical transplantation, which starts with at least a 6/12 match.

In a different scenario, a recent report shows that not only the traditional matching of HLA class I sequences in the peptide-binding region could drive the risk of GVHD or even impact the results of the transplantation. A dimorphism in HLA-B exon 1, which produces two different leader peptides, could provide information regarding relapse and non-relapse mortality risk after UCB transplantation. This confirms the importance and potential of high-resolution HLA typing in UCB graft selection and transplantation (23).

Current HLA-matching protocols could be unbalanced if a comparison is pretended between UCB and haploidentical transplantation clinical outcomes. A low- and intermediate-resolution HLA typing is enough for the latter case, due to the inherent relation between donor and patient. On the other hand, and according to the previously mentioned evidence, allelic-level HLA typing would be more appropriate in a non-related UCB setting, not only for HLA-DRB1 but for all HLA genes. There is a potential improvement of the UCB procedure in this area, which could lead to the obtention of better clinical outcomes if additional characteristics are included in the donor selection process.

## Outcomes of Haploidentical Transplantation vs. Umbilical Cord Blood

### Pediatric Patients

Although the reduction trend in UCB use aligns with the haploidentical transplantation increase, there are not many studies comparing both HSPC sources in terms of clinical efficacy. In pediatric patients, only two publications were found (24, 25) while in adults, including some studies with double cord transplant, there are five published studies (26–30), all of them in malignant disease, retrospective, and non-randomized. Recent work published a comparison of pooled data from these trials in terms of GVHD and relapse incidence, non-relapse-associated mortality, and 2-year disease-free survival (DFS), in which no statistically significant differences were found between the two sources (31). The obtention of conclusive results from these comparisons is a complicated task, mainly because of patient heterogeneity in terms of disease stages, previous treatments, conditioning regimen, conditioning intensity, GVHD prophylaxis, and variability in clinical outcomes definition.

However, pediatric UCB-transplanted patients showed a statistically significant (<0.001) delayed neutrophil recovery (determined as the first out of 3 consecutive days with absolute count ≥ 0.5 × 10^9^/L) in both studies, with a median time of 20 and 16 days compared to haploidentical transplanted patients, with a median time of 13 days in both reports (29, 30). Both studies had identical endpoint definitions, all patients had malignant diseases (acute lymphoblastic leukemia and acute myeloid leukemia), although there were some differences in the conditioning regimen and GVHD prophylaxis treatment for all groups (24, 25).

### Adult Patients

Published studies in adults exhibited less consistent results. The median time of neutrophil reconstitution was significantly different in three out of five papers comparing UCB and haploidentical transplantation (T-cell repleted) (26, 29, 30). The neutrophil median recovery time was 21 days for UCB and 18 days for haploidentical transplantation. The median time of platelet reconstitution was only reported in three studies (27, 28, 30), and there is an observable delay in UCB (41 and 38 days) compared to haploidentical transplantation (27 and 24 days). However, it is not possible to determine if these differences may be impacting any of the clinical outcomes, such as overall survival, PFS, or GVHD incidence, evaluated by Li et al. (31). Infections were reported by some authors as the cause of transplant-related death independently from the graft source (26, 29). Interestingly, there were no differences in the incidence of infections between both groups, although the neutrophil and platelet reconstitution delay found in UCB may lead to the longest hospitalization times, more transfusions, and a higher probability of complications (27). A recent meta-analysis and systematic review of haploidentical and non-related UCB donor transplantation of a relatively large population of adult and pediatric patients found no differences when comparing chronic GVHD incidence and DFS at 2 years but found a statistically significant higher risk for acute GVHD in the haploidentical adult transplantation group (32).

The website www.clinicaltrials.gov was consulted to verify if any ongoing clinical trial was comparing outcomes of transplantation from haploidentical and non-related UCB donors. Three interventional, open-label studies were found, two for hematological malignancies and one for ß-thalassemia. The latter lacks information since 2015 and its current state is unknown. The studies in hematological malignancies were designed to compare in parallel groups double UCB vs. haploidentical transplantation. One of those was terminated due to slow accrual—with only two enrolled patients—while the third one, a phase III active study with 368 enrolled patients, was recently completed and published (33). This is the only non-retrospective, randomized, and stratified study found so far comparing these two HSPC sources for transplantation. The study was performed in adults from 18 to 70 years of age, with high-risk acute leukemia or lymphoma (Hodgkin or no-Hodgkin) and two sources of HSPC available: first, two UCB units matched at least 4/6 each one with a cell dose > 1.5 × 10^7^ and second, a haploidentical bone marrow donor matched for at least one allele per loci (HLA-A, -B, -C, DRB1) at allele-level. After randomization, 172 patients received double-UCB transplantation, and 153 patients were transplanted from a haploidentical donor in a post-transplant cyclophosphamide setting. All patients received a reduced intensity conditioning regimen (33).

As opposed to the previously mentioned studies, the median time of neutrophil recovery in this trial was lower for double UCB (15 days) than for haploidentical donors (17 days) and is even statistically significant when comparing the cumulative incidence at day 56, although it is not completely clear if this variable assessment is identical to the previous studies mentioned. Platelet recovery, however, remains slower in double UCB (median 42 days) than in haploidentical donors (median 28 days). As for GVHD, there were no significant differences between treatments either in the acute or chronic frame. There were no statistically significant differences between the two treatments in the probability of 2-year PFS (primary endpoint) and 2-year incidence of relapse/progression; nonetheless, the 2-year incidence of non-relapse mortality was significantly higher in double-UCB-transplanted patients, which leads to a lower 2-year overall survival than the haploidentical graft receptors (secondary endpoints). In this case, the most common cause of death in both groups was a recurrent disease, followed by infection. Although no analysis regarding this data is provided there is a difference of only 2% in deaths proportion by infection in UCB transplantation compared to haploidentical (9.15 vs. 7.1%) (33).

## Discussion

To determine which is the most favorable donor alternative between unrelated UCB and haploidentical donors for pediatric and adult HSPC transplantation candidates with any hematological disease is not an easy task, considering the diversity of the results. To the best of our knowledge, there are no published comparisons between the two sources in the treatment of non-malignant diseases. There is also an unexplored possibility comparing haploidentical transplantation with a higher matching UCB donor, considering four HLA loci in high-resolution typing, a condition that may improve the neutrophil reconstitution time, as described by Eapen et al. (18). It is possible that upgrading the matching of UCB units with the patient leads to a better clinical outcome in terms of immune reconstitution and consequently in overall survival or PFS even in the setting of haplo-cord transplantation.

Although there is not enough evidence favoring haploidentical over UCB transplantation from the clinical perspective, there is an increase of haploidentical transplantation vs. UCB (10, 13, 14). This trend may not only be caused by the higher possibility of finding a suitable donor and the lower costs but also because the neutrophil engraftment delay could turn into early complications and longer hospitalization care. It is not known whether the UCB scenario globally represents higher costs for health services compared to haploidentical transplantation, or if the latter might imply even higher long-term costs considering the required treatment for chronic GVHD. More research is required to improve and expand possibilities of HSPC graft source for patients that do not have an identical family donor.

Finally, it is important to note that just as haploidentical transplantation has improved over time, UCB transplantation may reach better results upgrading the clinical criteria and practice, especially because it has gone through a shorter path in history. Even though the comparisons so far have not been equitable between both sources in terms of HLA matching, which is one of the main success indicators, the lack of statistically significant differences in clinical outcomes strongly suggests that improving the UCB donor selection could definitively raise a new scenario for this source, adding possibilities for those patients lacking a related donor and joining forces with haploidentical transplantation possibilities. It is possible that the reduction of UCB use as an HSPC source compared to the haploidentical donor is not essentially based on research data, but it could be also driven by other conditions, such as the knowledge and skills in the clinical practice in some countries, economic issues, and difficulties of finding a donor.

## Author Contributions

DV and A-MP-A contributed to the initial conception of comparing the two sources for transplantation reviewing the available bibliography. LN-Q, MC, ME, and BC-R contributed with the clinical perspective for comparison. DV wrote the first draft of the manuscript, A-MP-A wrote sections of the manuscript. BC-R had financial support. All authors contributed to manuscript revision, read, and approved the submitted version.

## Funding

This work was supported by the Sistema General de Regalías. Fondo de CTeI, Colombia and Fondo Financiero Distrital de Salud (FFDS). Secretaría Distrital de Salud, Bogotá DC, Colombia. BPIN: 2016000100035.

## Conflict of Interest

The authors declare that the research was conducted in the absence of any commercial or financial relationships that could be construed as a potential conflict of interest.

## Publisher's Note

All claims expressed in this article are solely those of the authors and do not necessarily represent those of their affiliated organizations, or those of the publisher, the editors and the reviewers. Any product that may be evaluated in this article, or claim that may be made by its manufacturer, is not guaranteed or endorsed by the publisher.
