# Supplementary figures and images for: Clinical Outcomes of Unrelated Umbilical Cord Blood Graft vs. Haploidentical Donor Transplantation: Critical Issues for an Adequate Comparison
Source: Front Med (Lausanne). 2021 Oct 28;8:749810. doi: 10.3389/fmed.2021.749810 (PMC8581238; doi:10.3389/fmed.2021.749810)

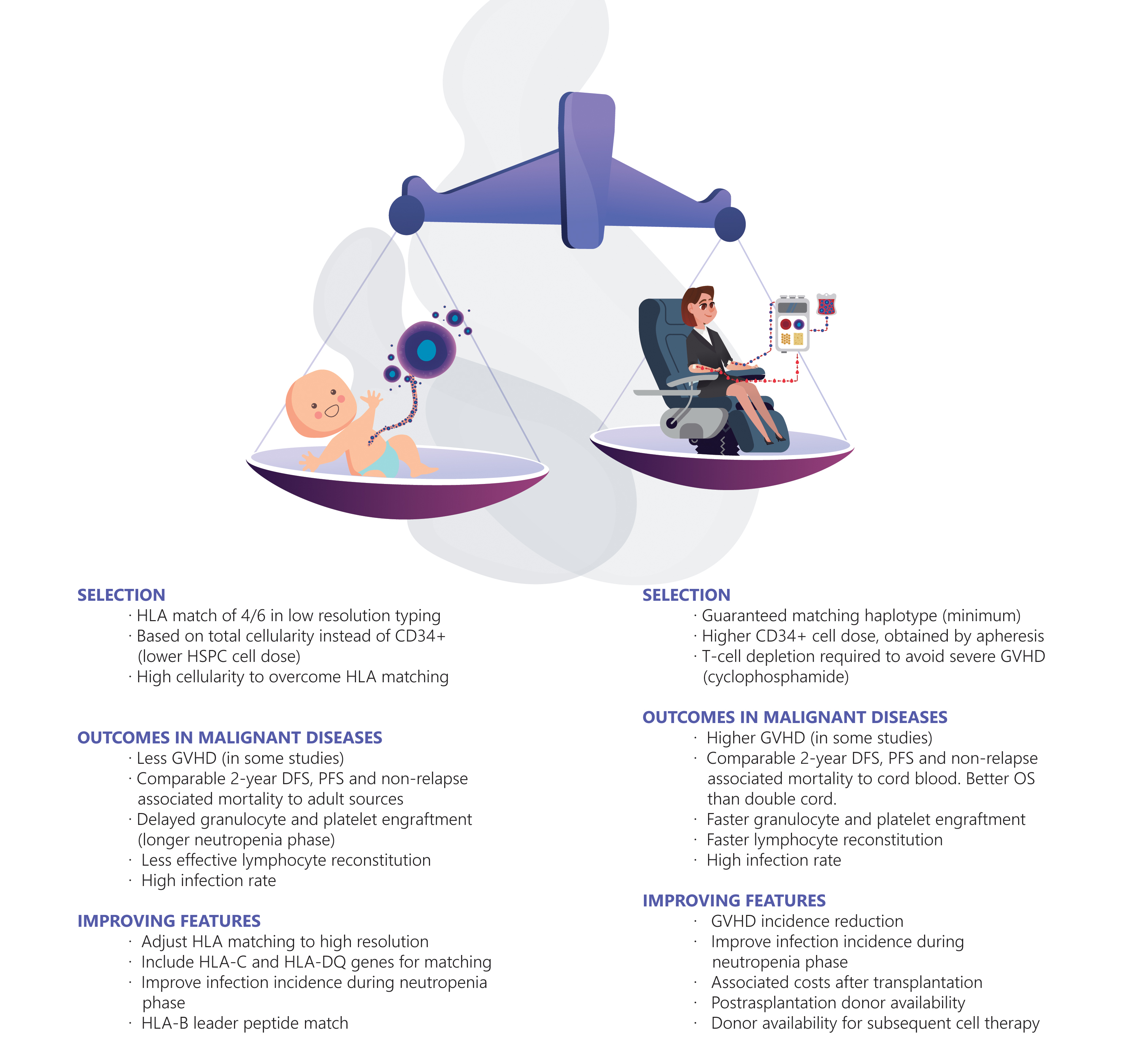

Supplement: Supplementary file 1 [file Image_1.JPEG]
